# Algorithmic scholarship and academic evaluation: governance misalignment in the age of generative AI

**DOI:** 10.3389/frai.2026.1862980

**Published:** 2026-07-08

**Authors:** Cristina Baciu, Gopalakrishnan Mohan, Jeffrey Wilson

**Affiliations:** W. P. Carey School of Business, Arizona State University, Tempe, AZ, United States

**Keywords:** academic evaluation, authorship, distributed cognition, generative artificial intelligence, higher education, research integrity, scholarly governance

## Abstract

**Introduction:**

Generative artificial intelligence (AI), particularly large language models, is rapidly becoming embedded in academic research and scholarly publishing. These systems assist with drafting, literature synthesis, and analytical writing, increasingly contributing to the production of academic text. This shift raises a central question: how should higher education institutions evaluate scholarly contribution when parts of research production become technologically mediated?

**Methods:**

This paper examines governance misalignment between publication standards and institutional evaluation systems in higher education. Drawing on an exploratory qualitative survey of 18 journal editors and associate editors across business-related disciplines, we analyze editorial perspectives on AI-assisted manuscript preparation, authorship, accountability, productivity, and academic evaluation.

**Results:**

We identify three recurring concerns: policy fragmentation, ambiguity surrounding authorship and accountability, and apprehension about AI-enabled productivity acceleration. We introduce the concept of *metric distortion* to describe the weakening relationship between measurable scholarly outputs and the intellectual labor those outputs are assumed to represent under conditions of AI-assisted production. As drafting and textual production become more technologically scalable, publication-based indicators may become less reliable proxies for conceptual contribution and interpretive effort.

**Discussion:**

Building on scholarship on academic capitalism, audit culture, and digital governance, the paper argues that generative AI functions as a stress test for existing evaluation regimes. We propose a process-based framework emphasizing disclosure, documented intellectual contribution, and institutional alignment between editorial governance and tenure evaluation systems.

## Introduction

Generative artificial intelligence (AI), particularly large language models (LLMs), is rapidly becoming embedded in academic research practice. These tools assist with drafting text, synthesizing literature, generating code, and refining analytical writing. Unlike earlier computational technologies that primarily supported data processing or information retrieval, LLMs participate directly in the production of scholarly text and argument. Their diffusion represents more than a technical improvement; it signals a structural shift in how research is generated and presented. Recent empirical work suggests that this shift is already visible in scholarly publishing, with large-scale analyses detecting substantial growth in LLM-assisted writing across scientific abstracts (Kobak et al., 2025).

Generative AI also extends forms of distributed cognition within scholarly work. Research production increasingly unfolds across human actors and technological systems that share aspects of drafting, synthesis, and organization. Yet while epistemic processes may become distributed, accountability for scholarly claims remains attached to identifiable human agents. This tension aligns with recent work arguing that generative AI complicates scientific authorship by making scholarly production more relational while leaving responsibility, verification, and interpretive authority with human researchers (Watson et al., 2025).

Higher education institutions are especially exposed to the implications of this shift because academic evaluation systems remain heavily output-oriented. Tenure and promotion processes frequently rely on publication counts, journal rankings, authorship conventions, and citation metrics as proxies for intellectual contribution. This reliance reflects broader transformations in higher education governance associated with academic capitalism and performance-based accountability regimes (Slaughter and Rhoades, 2004; Shore and Wright, 2000). As universities have adopted increasingly managerial approaches to evaluation, measurable outputs have become institutional currencies of merit (Espeland and Sauder, 2007; Power, 1997). In such environments, productivity functions simultaneously as an indicator, an incentive, and a signal of scholarly value.

The integration of generative AI into research workflows complicates these evaluative logics. If drafting, synthesis, and textual production become more technologically scalable, the relationship between output volume and intellectual labor may weaken. Publication counts and stylistic fluency may no longer reliably signal conceptual originality or interpretive depth. At the same time, journals and publishers have begun developing policies governing AI use in manuscripts, including disclosure requirements and restrictions on AI-generated content, while universities have been slower to update tenure and promotion frameworks to account for AI-mediated scholarship. This divergence suggests a growing governance gap between editorial regulation and institutional evaluation systems.

Recent publisher and journal policies further illustrate this fragmentation. While most prohibit AI authorship and require disclosure, they diverge substantially in what constitutes acceptable use, ranging from strict prohibitions on AI-generated drafting to conditional acceptance with citation and documentation requirements. Recent analyses of publisher and journal guidance similarly show substantial variation in disclosure requirements, allowable uses, and the relationship between publisher-level and journal-level policies (Ganjavi et al., 2024; Perkins and Roe, 2024). This variability introduces additional uncertainty for faculty navigating publication and evaluation systems.

Much of the emerging literature on generative AI in higher education has focused on teaching, academic integrity, and classroom assessment. Comparatively less attention has been given to how generative AI may reshape institutional systems of scholarly evaluation. Existing discussions of AI-assisted research often emphasize plagiarism detection, disclosure practices, authorship boundaries, or technical governance concerns (Ganjavi et al., 2024; Perkins and Roe, 2024; Resnik and Hosseini, 2025). However, fewer studies examine how generative AI may destabilize the evaluative assumptions underlying tenure and promotion systems, particularly those that rely heavily on publication-based indicators of scholarly contribution.

Recent scholarship has also begun documenting the growing role of generative AI in research workflows and scholarly publishing (Bearman et al., 2023; Else, 2023; Nature, 2023). These developments raise broader governance questions concerning authorship, accountability, and the meaning of intellectual contribution under conditions of technologically mediated production. This study addresses that gap by examining how journal editors interpret the implications of AI-assisted scholarship for academic evaluation systems.

Journal editors occupy a critical governance position within academic knowledge production. They establish publication standards, interpret authorship norms, and oversee peer review processes that shape the credibility of scholarly outputs. Because editors operate at the intersection of research production and evaluation, their perspectives offer early insight into how generative AI is reshaping expectations for authorship, accountability, and scholarly contribution.

To examine these issues, this paper focuses on journal editors and associate editors in business-related disciplines, where publication metrics play a particularly prominent role in academic careers. Editors play a central role in interpreting publication standards and responding to emerging technologies that affect scholarly communication. Their perspectives therefore provide a valuable window into how AI use is currently being negotiated within academic gatekeeping practices.

We pursue three questions. How are journals currently governing the use of generative AI in manuscript preparation and authorship? How do editors perceive the implications of AI-assisted research production for accountability and evaluation in output-oriented academic systems? And what do these emerging governance practices reveal about metric distortion and misalignment between editorial standards and institutional tenure and promotion frameworks? In answering these questions, we conceptualize generative AI as a stress test for metricized evaluation regimes and develop a process-based framework for aligning publication governance with academic evaluation in higher education.

This paper makes three contributions to the literature on higher education governance. First, it conceptualizes generative AI as a potential source of metric distortion within performance-based evaluation systems, specifying how AI-mediated acceleration of textual production can decouple visible outputs from underlying intellectual labor. Second, it provides early empirical evidence of governance uncertainty among journal editors regarding AI-mediated scholarship, highlighting emerging tensions between editorial policy development and institutional evaluation systems. Third, it proposes a process-based framework for evaluating scholarly contributions that emphasizes disclosure, documented intellectual engagement, and alignment between editorial governance and tenure policies.

Rather than framing generative AI as either threat or panacea, this paper situates these technologies within broader transformations in academic labor and performance regimes. The central challenge for higher education is not whether AI will be used in research—that transition is already underway—but how evaluation systems can adapt without intensifying inequities or undermining confidence in scholarly contribution.

## Literature review: academic evaluation, metricization, and governance under generative AI

### Academic capitalism and the governance of research productivity

Over the past three decades, higher education governance has shifted toward performance-oriented models that emphasize accountability, competition, and measurable output (Slaughter and Rhoades, 2004). Within academic capitalism, research productivity functions not only as an intellectual contribution but also as institutional currency. Publications—especially those appearing in high-status journals—serve as key indicators of academic value.

This transformation is closely tied to the rise of audit culture (Power, 1997; Shore and Wright, 2000). Universities increasingly rely on quantitative metrics to evaluate academic work. Common indicators include publication counts, citation indices, journal impact factors, and grant income. These measures do more than assess performance; they shape academic behavior.

Research on evaluation systems shows that metrics produce reactivity. When institutions measure performance in specific ways, scholars adjust their behavior accordingly (Espeland and Sauder, 2007). In academic environments, research topics, collaboration patterns, and publication strategies often align with evaluation criteria.

Digital infrastructures have intensified this metric orientation. Research evaluation is now embedded in broader systems of digital governance (Williamson, 2017; Gulson et al., 2022). Citation analytics platforms and research dashboards provide continuous visibility into scholarly productivity, increasing the transparency and comparability of academic output.

### Metricization and the fragility of proxies

Output-based evaluation systems rely on a core assumption: measurable indicators correlate with intellectual contribution. Publication counts, citation impact, and journal prestige function as proxies for qualities such as originality, rigor, and interpretive depth. While imperfect, these proxies have historically been treated as workable approximations of scholarly labor.

However, metric-based governance also creates unintended consequences. Scholars may adapt strategically to evaluation systems by dividing research into smaller publications, expanding co-authorship networks, or prioritizing topics that generate rapid output (Espeland and Sauder, 2007; Muller, 2018). Metrics influence behavior precisely because they function as signals of value.

Generative AI introduces a new complication to this dynamic. Unlike earlier research technologies—such as statistical software or bibliographic databases—large language models operate directly within the domain of scholarly writing. They assist with drafting, summarizing literature, structuring arguments, and refining language. As a result, parts of the research production process can become faster and more efficient. This acceleration is consistent with broader concerns that AI-enabled scientific productivity may create an “illusion of understanding,” in which researchers produce more while potentially weakening the relationship between output, comprehension, and interpretive depth (Messeri and Crockett, 2024).

We describe the implications of this shift as *metric distortion*. Metric distortion occurs when the relationship between measurable outputs and the intellectual labor they represent weakens. AI-assisted drafting does not necessarily reduce research quality, but it may reduce the time and effort required to produce polished manuscripts. When production becomes more efficient, publication counts may become less reliable indicators of conceptual depth or originality. The empirical findings presented later in this study suggest that concerns associated with metric distortion are already emerging within editorial governance practices, particularly in discussions surrounding AI-assisted drafting and accelerated manuscript production.

Metric distortion differs from related concerns such as Goodhart’s Law, Campbell’s Law, and metric fixation. Those frameworks describe how indicators can become corrupted when they become targets for optimization (Campbell, 1979; Muller, 2018). Metric distortion instead refers to structural changes in the relationship between scholarly labor and measurable output under conditions of AI-assisted production. The concern is not simply strategic gaming of indicators, but the possibility that traditional evaluation metrics may no longer correspond as closely to the intellectual work they are assumed to represent.

### Digital governance and AI in higher education

Generative AI must also be understood within broader transformations in digital governance. Data-driven technologies increasingly shape research analytics, institutional benchmarking, and performance management in universities (Williamson, 2017; Gulson et al., 2022). These technologies influence how institutions monitor productivity and allocate resources.

Recent scholarship also emphasizes the broader opportunities and governance challenges associated with large language models in educational and research settings (Kasneci et al., 2023; Khalifa and Albadawy, 2024; Resnik and Hosseini, 2025). These studies highlight both the productivity potential of generative AI and the governance concerns associated with AI-assisted writing, evaluation, and accountability in higher education (Tlili et al., 2023).

Most early discussions of AI in higher education have focused on teaching and academic integrity. More recent work highlights governance and assessment challenges, particularly regarding transparency, accountability, and institutional oversight (Bearman et al., 2023). These concerns are especially relevant for tenure and promotion systems that rely on visible research outputs as evidence of scholarly merit.

Generative AI therefore functions as a stress test for existing evaluation regimes. It exposes how strongly academic assessment depends on stable relationships between visible output and intellectual labor. As textual production becomes more technologically scalable, universities may need to reconsider whether traditional evaluation indicators remain adequate.

Generative AI has rapidly entered scholarly publishing workflows. Large language models are increasingly used to assist with drafting, language editing, literature synthesis, and manuscript preparation. These developments have prompted growing concern within academic publishing regarding transparency, disclosure, authorship attribution, and accountability (Ganjavi et al., 2024; Nature, 2023; Perkins and Roe, 2024; Resnik and Hosseini, 2025).

Recent studies suggest that AI-assisted writing may alter editorial and peer-review practices by increasing the speed and volume of manuscript production while complicating traditional assumptions about scholarly contribution (Bearman et al., 2023). Editors and publishers are therefore emerging as key governance actors in determining how AI-generated or AI-assisted content should be evaluated within scholarly communication systems.

At the same time, institutional responses remain uneven. Some publishers have adopted formal disclosure requirements and explicit authorship restrictions, while others continue to rely on informal guidance. These developments raise broader questions about how editorial governance and institutional evaluation systems will adapt to AI-mediated research production.

This study builds on these emerging debates by examining how journal editors interpret the implications of generative AI for academic evaluation systems and scholarly accountability.

### Governance fragmentation and institutional lag

Institutional responses to technological change rarely occur simultaneously across governance levels. Higher education systems are decentralized, and journals, publishers, departments, and universities operate under different regulatory logics (Power, 1997). Policy adaptation often lags behind technological diffusion.

Editorial boards have begun introducing guidelines on AI use in manuscript preparation, often requiring disclosure of AI assistance and prohibiting listing AI systems as authors. Yet many tenure and promotion frameworks still lack clear guidance on AI-assisted scholarship.

This divergence creates governance misalignment between publication standards and institutional evaluation systems. Faculty may face inconsistent expectations across journals and tenure committees. Differences in access to AI tools and institutional support may also amplify existing inequalities in research productivity (Ivancheva, 2020).

In this context, generative AI represents not only a technological innovation but also a governance challenge. Universities must reconsider how intellectual contribution is documented, evaluated, and rewarded within digitally mediated research environments.

## Methods

### Research design

This study uses an exploratory qualitative design to examine how generative AI is interpreted and governed within scholarly publishing. Large language models are spreading quickly, and institutional policies are still evolving; an exploratory approach allows us to identify emerging governance patterns and areas of uncertainty.

The study focuses on journal editors and associate editors as key governance actors. Editors interpret publication standards, oversee peer review, and shape authorship expectations. Their perspectives provide insight into how AI use is currently being negotiated within scholarly gatekeeping.

### Sample and recruitment

Participants were faculty members serving as editors or associate editors for peer-reviewed journals in business-related disciplines, including management, marketing, finance, economics, agribusiness, and related areas. We selected these disciplines because they operate within strong publication cultures and well-established output-based tenure systems, making them a useful setting for examining how AI-mediated research may affect academic evaluation.

We identified editors through journal websites and professional networks and contacted them by email with an invitation to participate in the study. Participation was voluntary, and all responses were anonymized. Data collection occurred between September 2025 and January 2026.

A total of 67 editors and associate editors were contacted during recruitment, resulting in 18 completed responses. Although modest in size, the sample was sufficient for identifying recurring governance concerns and thematic patterns across editor perspectives. The sample was designed to capture a range of editorial perspectives across publication-intensive disciplines where research productivity strongly shapes career advancement. Although exploratory in scope, the sample included editors from journals differing in publication models, impact profiles, and institutional contexts. Consistent with qualitative inquiry, the goal was not statistical representativeness but interpretive insight into emerging governance tensions associated with AI-assisted scholarship. Although the sample is not statistically representative of all disciplines, it provides insight into governance perspectives within fields where publication metrics strongly influence career advancement.

### Data collection

Data were collected using a structured online questionnaire that included both closed-ended and open-ended questions. The survey asked editors about: the existence and content of journal policies governing AI use, perceived benefits and risks of generative AI in manuscript preparation, expectations regarding disclosure and authorship, and concerns related to peer review, productivity, and accountability. Open-ended questions allowed participants to describe emerging governance challenges in their own words. Responses were de-identified prior to analysis.

Example questions included: “Does your journal currently have a formal policy regarding AI-assisted manuscript preparation?”; “What concerns, if any, do you have regarding AI-assisted scholarly writing?”; “How should responsibility for AI-generated content be handled in academic publishing?”; “Do you believe generative AI may affect how scholarly productivity is evaluated in tenure and promotion systems?”; and “Should AI-assisted content require formal disclosure during submission?” A summary of the questionnaire structure is provided in Appendix.

### Data analysis

We analyzed qualitative responses using an iterative thematic coding approach (Braun and Clarke, 2006). In the first stage, we conducted open coding to identify recurring concerns related to policy fragmentation, authorship ambiguity, and productivity acceleration. In the second stage, we refined these codes into broader categories that reflected institutional misalignment and evaluation risk.

Coding proceeded in multiple stages. First, responses were reviewed independently to identify recurring concepts related to governance, authorship, accountability, and productivity. Initial open codes were then compared and refined collaboratively among all three authors. Through iterative discussion, related codes were grouped into broader thematic categories reflecting institutional misalignment and evaluation strain.

Disagreements regarding thematic interpretation were resolved through discussion and re-examination of the original responses. The analysis prioritized conceptual consistency and interpretive coherence rather than statistical reliability measures, consistent with exploratory qualitative inquiry.

The goal of the analysis was interpretive rather than statistical. Closed-ended responses were summarized descriptively to provide context for the qualitative findings, but the study does not attempt to estimate the prevalence of views across disciplines. Instead, it identifies governance tensions that may inform institutional policy discussions.

### Limitations

Several limitations should be acknowledged. First, the study draws on a relatively small sample of editors and associate editors within business-related disciplines. Although appropriate for exploratory qualitative inquiry, the sample does not capture the full diversity of governance perspectives across academic fields, institutional types, or publishing contexts. Different disciplines may experience AI-assisted scholarship and evaluation pressures in substantially different ways.

Second, the study relies on self-reported perceptions rather than direct observation of editorial decision-making or institutional evaluation practices. Editors’ responses therefore reflect interpretive judgments and anticipatory governance concerns rather than fully institutionalized practices. As generative AI technologies continue to evolve, these perceptions may also shift over time.

Third, the study does not incorporate triangulation through institutional documents, observational data, or comparative analysis of formal editorial policies. Future research could strengthen the empirical foundation of this work by combining interview or survey data with policy analysis, manuscript review practices, or longitudinal institutional evidence.

Finally, the study captures an early stage of institutional adaptation to generative AI. Many editorial policies and university governance frameworks remain under development. The findings should therefore be interpreted as indicative of emerging governance tensions rather than definitive evidence of long-term institutional transformation.

Despite these limitations, the study provides timely insight into how journal editors interpret the implications of generative AI for scholarly evaluation systems. It offers an exploratory empirical foundation for future research on governance, accountability, and AI-mediated academic production.

### Findings

Across all three themes, editors described growing uncertainty about how existing governance frameworks apply to AI-assisted scholarship. Some responses emphasized opportunity and efficiency, while others stressed accountability risks and evaluation instability. This variation is itself analytically important. It suggests that institutional responses to generative AI remain unsettled and uneven across scholarly communities.

Journal governance is beginning to adapt through disclosure policies and evolving authorship standards. Institutional tenure frameworks, however, remain comparatively underdeveloped. The findings therefore indicate that governance misalignment extends beyond editorial policy and into the broader structure of academic evaluation systems.

### Policy fragmentation and uneven regulatory development

Editors reported substantial variation in how journals are responding to generative AI. Some journals have adopted formal policies requiring authors to disclose the use of AI and clarifying that AI systems cannot be listed as authors. Other journals are still developing policies and addressing AI use on a case-by-case basis.

Several editors described uncertainty about whether formal policies could keep pace with technological change. One respondent explained:

“We are trying to establish standards while the technology itself is evolving almost monthly.”

Another editor emphasized disciplinary inconsistency:

“Some journals are moving aggressively on disclosure policies, while others still have no formal guidance at all.”

These responses suggest that governance fragmentation is not simply procedural inconsistency. It also reflects uncertainty about how rapidly editorial standards should adapt to evolving AI capabilities.

Several editors emphasized that practices remain inconsistent even within the same discipline. One editor explained, “*We have issued guidance, but interpretation* var*ies widely across submissions*.” Another noted, “*The technology has moved faster than our formal policies*.”

This variation reflects the decentralized governance structure of scholarly publishing (Power, 1997) but also creates uncertainty for authors whose work is evaluated by both journals and tenure committees. When editorial expectations differ and institutional guidelines remain unclear, faculty face ambiguous standards regarding acceptable AI use. From a governance perspective, this fragmentation signals institutional lag in responding to technological change.

### Ambiguity in authorship and accountability

Editors also described uncertainty about authorship boundaries in AI-assisted research. Most respondents agreed that human authors must remain accountable for their work and that AI systems should not be credited as authors. However, editors struggled to identify where assistance becomes delegation.

Although most editors agreed that human authors should remain accountable, responses differed regarding acceptable levels of AI assistance. Some viewed AI primarily as an advanced editorial tool, while others worried that generative systems increasingly participate in intellectual production itself.

One editor noted: “Using AI for grammar correction feels very different from using it to draft an argument.”

Another observed: “At some point, the distinction between assistance and authorship becomes difficult to define operationally.”

These differences suggest that existing authorship norms may no longer map cleanly onto AI-mediated research workflows.

Several editors raised questions such as: “*How much drafting assistance is too much*?” and “*If AI suggests structure or phrasing, where does authorship responsibility begin and end*?” Some editors also expressed concern about verification, noting that AI-generated references and summaries can appear credible even when they contain errors. Editors, therefore, questioned whether authors are consistently validating AI-generated material.

These concerns reveal a potential vulnerability in output-based evaluation systems. Tenure frameworks assume that named authors represent the intellectual substance of their publications. When parts of textual production are technologically mediated, the relationship between visible authorship and underlying scholarly labor becomes less transparent. Editors’ uncertainty reflects strain within existing authorship conventions rather than isolated ethical violations.

### Productivity acceleration and metric distortion

The third theme involved concern about AI-enabled productivity acceleration. Editors acknowledged the benefits of AI tools, especially for language editing and literature organization, but many expressed concerns that generative tools lower the barriers to producing submission-ready manuscripts.

Editors differed in how they interpreted the implications of accelerated manuscript production. Some viewed AI-assisted drafting as a manageable extension of existing writing technologies. Others worried that increased submission volume could overwhelm editorial review systems.

One editor commented: “The issue is not necessarily quality decline. It’s that the volume of polished submissions could increase dramatically.”

Another warned: “We may eventually lose the ability to distinguish effort from efficiency.”

These responses suggest that concerns about productivity acceleration extend beyond individual misconduct. Editors frequently framed the issue as a structural challenge for evaluation systems that rely heavily on visible output indicators.

One editor remarked, “*The barrier to producing a readable draft is lower than ever*.” Another warned, “*We may see an increase in submissions that are polished but thin in conceptual contribution*.”

These observations point to the possibility of metric distortion in output-based evaluation regimes. The concept of metric distortion emerged interpretively from recurring editor concerns about the changing relationship between visible scholarly output and underlying intellectual effort. Editors repeatedly questioned whether publication counts and polished manuscripts would continue to function as reliable indicators of conceptual contribution under conditions of AI-assisted production.

One editor observed: “The concern is not simply that AI helps people write faster. It’s that our existing evaluation signals may stop meaning what we think they mean.”

Another explained: “If everyone can produce polished drafts much more quickly, then publication volume alone becomes a much weaker indicator of contribution.”

These responses illustrate how editors linked AI-enabled productivity acceleration to broader concerns about the stability of evaluation proxies.

If AI-assisted drafting reduces the time required to produce publishable text, publication counts may become weaker indicators of intellectual contribution. In fields where tenure decisions depend heavily on visible productivity, accelerated output may increase pressure to prioritize quantity over conceptual depth.

Importantly, editors did not frame productivity acceleration primarily as misconduct. Instead, they described structural concerns. When textual fluency becomes technologically scalable, traditional evaluation indicators may lose some of their ability to distinguish levels of scholarly effort. In this sense, generative AI functions less as an ethical anomaly and more as a stress test for evaluation systems built on output-based proxies.

Across all three themes, editors highlighted policy inconsistency, accountability uncertainty, and growing strain on traditional evaluation indicators. Journal governance is beginning to respond to generative AI, but institutional tenure frameworks remain largely unchanged. The findings point to a broader governance misalignment that extends beyond editorial policies to the structure of academic evaluation itself.

## Discussion

The findings suggest that generative AI functions as more than a new research tool within academic work. Editors described policy fragmentation, uncertainty about authorship, and concerns about accelerated research production, revealing strain in governance systems that rely heavily on output-based indicators. When editorial standards evolve faster than tenure policies, faculty operates within overlapping but misaligned accountability structures, creating uncertainty about how scholarly contributions should be evaluated.

Importantly, metric distortion is not presented here as a purely abstract theoretical construct. Rather, it is an interpretive framework developed from recurring editor concerns regarding accelerated manuscript production, reduced barriers to polished textual output, and uncertainty about how scholarly effort should be evaluated under conditions of AI-assisted writing.

Editors repeatedly described scenarios in which visible productivity indicators might become increasingly detached from the labor traditionally associated with research production. The concept of metric distortion therefore captures both a governance concern identified empirically and a broader institutional challenge for output-based evaluation systems.

### Faculty-level implications of policy fragmentation

The policy fragmentation described by editors has direct implications for faculty research practice. As generative AI becomes more embedded in manuscript development, faculty are increasingly required to navigate a publication environment in which expectations vary across journals, publishers, and professional associations. Although a broad consensus has emerged that AI cannot be credited as an author and that human scholars remain fully accountable for submitted work, policies diverge substantially in what forms of AI use are permitted, when disclosure is required, and how such disclosure should be presented. Some publishers allow limited assistance with writing and editing, others require detailed disclosure and citation, and some take a more restrictive stance toward AI-generated drafting altogether. This variability creates a fragmented governance landscape that faculty must interpret strategically as part of their research and publication decisions.

One consequence of this fragmentation is strategic uncertainty. Faculty may reasonably ask whether the same AI-assisted practice that is acceptable in one journal would be viewed as inappropriate in another. Because many scholars submit across journals with different editorial rules, compliance becomes situational rather than standardized. Recent empirical work on AI-use disclosure similarly shows that editors and authors face blurred thresholds around when disclosure is necessary and what level of detail is sufficient (Lingard et al., 2025). This uncertainty is especially consequential for early-career faculty, who often operate under intense publication pressure and may be less willing to risk sanctions, reputational harm, or later disputes over disclosure sufficiency. In this context, AI use is not merely a matter of efficiency or innovation; it becomes a matter of professional judgment under conditions of regulatory inconsistency.

A second implication is the emergence of new compliance burdens. AI policies do not simply regulate authorship in the abstract; they often require scholars to document which tools were used, how they were used, where they influenced the manuscript, and whether their outputs were verified. In some cases, authors may also need to consider intellectual property implications, provide citations to AI-generated material, or conform to stage-specific disclosure requirements. These expectations add new layers of administrative and interpretive labor to the publication process. Rather than reducing workload in a straightforward way, generative AI may shift some labor from drafting into documentation, monitoring, and self-auditing.

Policy fragmentation may also exacerbate unevenness in access and capability. Faculty do not encounter generative AI under equal conditions. Access to paid tools, institutional guidance, training, and departmental norms varies considerably across universities and academic units. Some scholars may be supported in experimenting with AI-assisted workflows, while others may face ambiguity without training or may avoid these tools altogether for fear of violating unclear expectations. As a result, the benefits of generative AI are unlikely to be distributed evenly. Existing institutional inequalities may deepen if some faculty are better positioned than others to use AI effectively, responsibly, and in ways that remain legible to journal gatekeepers.

Finally, fragmented AI governance may reinforce metric pressure within output-oriented academic systems. If AI tools lower the time required to produce polished manuscripts, publication volume may rise without a corresponding change in how institutions evaluate scholarly merit. Yet because journal rules differ, faculty must pursue productivity gains while simultaneously managing compliance risks. This combination may intensify pressure rather than relieve it. Scholars may feel compelled to adopt AI in order to remain competitive, even as they operate in a policy environment that provides incomplete and inconsistent guidance. Under these conditions, generative AI does not simply accelerate research production; it alters the strategic terrain on which faculty negotiate visibility, legitimacy, and career advancement.

### Governance misalignment and institutional lag

Higher education governance is decentralized and multi-layered (Power, 1997). Journals, departments, and universities operate under different regulatory logics and timelines, and the diffusion of generative AI appears to have intensified this institutional lag. Editorial boards have begun introducing disclosure policies and clarifying authorship rules, while many tenure and promotion frameworks still lack guidance on AI-assisted scholarship.

This divergence creates uncertainty for faculty navigating academic careers. In academic capitalist environments, publication output functions as institutional currency (Slaughter and Rhoades, 2004), and career advancement depends heavily on measurable productivity. When editorial expectations vary and institutional evaluation systems remain unclear, scholars may face inconsistent standards. These findings suggest that governance misalignment may influence both research practices and academic career incentives.

### Metric distortion and evaluation fragility

The concept of metric distortion helps explain these developments. Metric distortion occurs when measurable outputs no longer correspond closely to the intellectual labor they are assumed to represent. Evaluation systems rely on output indicators because they function as practical proxies for scholarly effort and quality (Espeland and Sauder, 2007; Muller, 2018), but generative AI complicates this relationship. Tools that assist with drafting, summarizing, and organizing arguments reduce the time required to produce polished manuscripts, altering the conditions under which metrics were originally institutionalized.

This change does not imply a decline in research quality, but it introduces strain into evaluation systems designed for earlier production conditions. If publication output becomes easier to generate, traditional metrics may lose some of their ability to distinguish levels of intellectual contribution. Editors in this study did not describe widespread ethical violations; instead, they expressed uncertainty about how existing evaluation norms apply to AI-assisted work. At present, these tensions are best understood as emerging governance concerns rather than established institutional outcomes. The findings do not demonstrate that evaluation systems have already become destabilized. Rather, they indicate growing uncertainty about whether existing metrics will remain reliable under conditions of AI-assisted production.

### Digital governance and performance intensification

Generative AI should also be understood within broader processes of digital governance in higher education. Data-driven technologies increasingly shape research analytics, institutional benchmarking, and performance monitoring (Williamson, 2017; Gulson et al., 2022). Citation dashboards and analytics platforms provide continuous feedback on productivity, intensifying metric visibility and accelerating performance pressures.

As Espeland and Sauder (2007) demonstrate, metrics generate reactivity as scholars adapt their behavior in response to evaluation indicators. Generative AI may amplify this dynamic. When manuscript preparation becomes more efficient, scholars may increase submission volume, reinforcing incentives that favor quantity over conceptual depth in performance-driven environments.

### Equity and stratification implications

One possible implication of governance misalignment involves distributive consequences across institutions and career stages. Access to AI tools, training, and institutional support varies across universities, and faculty at well-resourced institutions may adopt generative technologies more quickly. Early-career and contingent scholars may face greater pressure to use AI-assisted production to remain competitive. In performance-oriented evaluation systems, these pressures can intensify existing inequalities (Ivancheva, 2020).

If evaluation criteria remain unchanged while AI adoption accelerates unevenly, productivity gaps may widen. Institutional silence regarding AI use functions as a policy choice that shapes incentives and redistributes advantage.

These implications were not discussed extensively by editors themselves and should therefore be interpreted as theoretical extrapolations informed by existing scholarship on academic inequality and performance regimes.

### Toward institutional alignment

**Figure 1:**
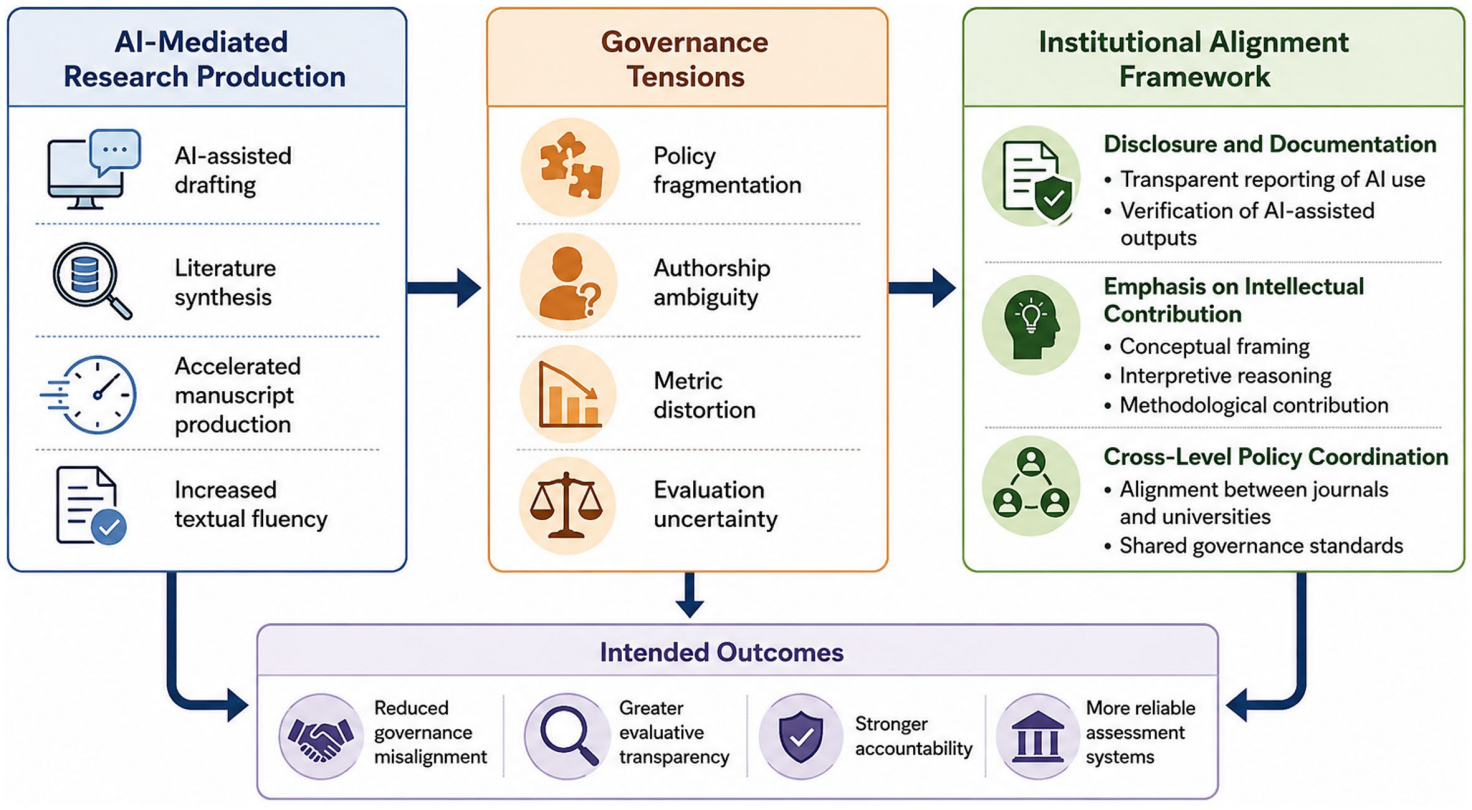
Governance misalignment and institutional alignment under generative AI. Conceptual model illustrating how AI-mediated research production generates governance tensions within academic evaluation systems and how process-based institutional alignment mechanisms may reduce governance misalignment.

The findings highlight the need for stronger coordination between editorial governance and institutional evaluation systems. The proposed framework is summarized in Figure 1, which illustrates how AI-mediated research production generates governance tensions within academic evaluation systems and how process-based institutional alignment mechanisms may reduce governance misalignment.

The proposed framework differs from traditional output-based evaluation approaches by emphasizing process-oriented documentation of scholarly contribution rather than relying exclusively on visible productivity indicators. Existing systems primarily assess outputs such as publication counts and journal prestige. The framework proposed here supplements those indicators with evidence regarding how research was produced, interpreted, and validated under conditions of AI-assisted scholarship.

Output-based evaluation does not need to be abandoned, but institutions may need to supplement quantitative indicators with clearer documentation of scholarly contribution. As generative AI reshapes research production, universities and journals must reconsider how scholarly work is documented, evaluated, and rewarded.

We propose three principles for institutional alignment.

1. *Disclosure and documentation*: Universities and journals should require clear documentation of how AI tools were used in research production and how outputs were verified, integrating this information into both editorial review and tenure dossiers.
2. *Emphasis on intellectual contribution*: Evaluation committees should give greater weight to conceptual framing, methodological design, and interpretive reasoning rather than relying solely on publication counts or journal prestige, explicitly recognizing that textual fluency is increasingly technologically mediated.
3. *Cross-level policy coordination*: Universities should work with journals and professional associations to align publication policies with tenure criteria, so that expectations regarding AI use are consistent across editorial standards, promotion guidelines, and professional norms.

These measures do not restrict AI-assisted research; instead, they support responsible integration while preserving accountability and fairness in evaluation systems.

Implementation would likely require coordination across multiple governance levels. Journals, universities, and professional associations would need to develop compatible disclosure standards and clearer expectations regarding AI-assisted research practices. Departments and tenure committees could operationalize this approach by inviting faculty to document AI use in research statements, clarify their intellectual contributions to AI-assisted work, and describe verification practices for AI-generated or AI-supported content, consistent with recent recommendations that researchers disclose, describe, and explain AI use while retaining responsibility for accuracy, bias, and interpretive judgment (Resnik and Hosseini, 2025). Although such reforms would require institutional adaptation, they may also improve transparency and preserve evaluative legitimacy under changing technological conditions.

The rapid diffusion of generative artificial intelligence is reshaping academic research practices. As this study shows, journal editors are already responding to policy fragmentation, authorship ambiguity, and concerns about AI-enabled productivity acceleration, while tenure and promotion systems have adapted more slowly. This gap reveals a growing governance misalignment between publication standards and institutional evaluation frameworks.

In output-oriented academic environments, measurable productivity functions as a proxy for intellectual contribution. Generative AI complicates this relationship: when drafting, synthesis, and textual production become more efficient, publication counts may no longer reliably reflect the effort required to produce scholarly work. The resulting metric distortion reflects structural strain within evaluation systems rather than widespread misconduct. The present study does not establish the long-term institutional effects of generative AI on academic evaluation. Rather, it identifies early governance tensions emerging within editorial and evaluative practices.

Institutional inaction has consequences. Faculty may encounter inconsistent expectations across journals and tenure committees, and uneven access to AI tools may amplify existing inequalities in research productivity. In this context, silence regarding AI use is not neutral; it shapes incentives and redistributes advantage within competitive evaluation regimes.

Universities face a strategic choice. They can continue to rely primarily on output-based metrics or supplement these measures with clearer documentation of scholarly contributions. Aligning editorial standards with tenure policies, requiring disclosure of AI use, and emphasizing interpretive and methodological work are practical steps toward reducing governance misalignment.

Generative AI will remain part of academic research. The key question is whether evaluation systems will evolve alongside these technological changes. Ensuring coherence between publication governance and institutional assessment will be essential for maintaining fairness, accountability, and trust in academic evaluation.

## Conclusion

This study examined how journal editors interpret the implications of generative AI for academic evaluation systems. Editors described growing uncertainty regarding authorship, accountability, disclosure practices, and productivity acceleration under conditions of AI-assisted scholarship. These concerns point toward an emerging governance misalignment between publication standards and institutional evaluation frameworks.

The paper contributes conceptually by introducing the idea of *metric distortion*. Metric distortion refers to the weakening relationship between measurable scholarly outputs and the intellectual labor those outputs are assumed to represent. The findings suggest that editors increasingly question whether traditional indicators of productivity will remain reliable under conditions of technologically accelerated research production.

At present, these concerns should be understood as emerging governance tensions rather than established institutional outcomes. The study does not demonstrate that generative AI has already destabilized academic evaluation systems. Rather, it identifies uncertainty regarding whether existing metrics and authorship conventions will remain adequate as AI-assisted research practices expand.

The study also contributes to broader discussions of academic governance and evaluation. It suggests that institutions may need to supplement output-based indicators with clearer documentation of scholarly contribution, disclosure practices, and evaluative standards for AI-assisted work. The proposed framework is intended not as a replacement for existing evaluation systems, but as a process-oriented supplement designed to preserve accountability under changing technological conditions.

Generative AI is likely to remain embedded in scholarly production. Future research should therefore examine how editorial governance, institutional policies, and faculty practices continue to evolve over time. Longitudinal and comparative studies across disciplines may be especially important for understanding how AI-mediated research production reshapes evaluation systems in higher education.

## Data Availability

The raw data supporting the conclusions of this article will be made available by the authors, without undue reservation.

## Appendix. Example survey questions

1. Does your journal currently have a formal policy regarding AI-assisted manuscript preparation?
2. What concerns, if any, do you have regarding AI-assisted scholarly writing?
3. How should responsibility for AI-generated content be handled in academic publishing?
4. Do you believe generative AI may affect scholarly productivity or evaluation systems?
5. Should AI-assisted content require formal disclosure during submission?
